# The landscape of PBMC methylome in canine mammary tumors reveals the epigenetic regulation of immune marker genes and its potential application in predicting tumor malignancy

**DOI:** 10.1186/s12864-023-09471-6

**Published:** 2023-07-18

**Authors:** A-Reum Nam, Min Heo, Kang-Hoon Lee, Ji-Yoon Kim, Sung-Ho Won, Je-Yoel Cho

**Affiliations:** 1 Department of Biochemistry, College of Veterinary Medicine, Seoul National University, 1 Gwanak-Ro, Gwanak-Gu, Seoul, 08826 Republic of Korea; 2 BK21 Plus and Research Institute for Veterinary Science, Seoul National University, Seoul, 08826 Republic of Korea; 3 Comparative Medicine Disease Research Center, Seoul National University, Seoul, 08826 Republic of Korea; 4 Interdisciplinary Program of Bioinformatics, College of Natural Sciences, Seoul National University, Seoul, 08826 Republic of Korea; 5 Department of Public Health Sciences, Graduate School of Public Health, Seoul National University, Seoul, 08826 Republic of Korea

**Keywords:** Methylome, Biomarker, Canine, PBMC, Mammary tumor, Immunity, Machine learning

## Abstract

**Background:**

Genome-wide dysregulation of CpG methylation accompanies tumor progression and characteristic states of cancer cells, prompting a rationale for biomarker development. Understanding how the archetypic epigenetic modification determines systemic contributions of immune cell types is the key to further clinical benefits.

**Results:**

In this study, we characterized the differential DNA methylome landscapes of peripheral blood mononuclear cells (PBMCs) from 76 canines using methylated CpG-binding domain sequencing (MBD-seq). Through gene set enrichment analysis, we discovered that genes involved in the growth and differentiation of T- and B-cells are highly methylated in tumor PBMCs. We also revealed the increased methylation at single CpG resolution and reversed expression in representative marker genes regulating immune cell proliferation (BACH2, SH2D1A, TXK, UHRF1). Furthermore, we utilized the PBMC methylome to effectively differentiate between benign and malignant tumors and the presence of mammary gland tumors through a machine-learning approach.

**Conclusions:**

This research contributes to a better knowledge of the comprehensive epigenetic regulation of circulating immune cells responding to tumors and suggests a new framework for identifying benign and malignant cancers using genome-wide methylome.

**Supplementary Information:**

The online version contains supplementary material available at 10.1186/s12864-023-09471-6.

## Background

Immune cells interact with the tumor and are involved in tumor invasion, metastasis, and systemic immune cell exhaustion in the tumor environment [1]. Accordingly, cancer treatments have been developed using immune checkpoint inhibitor (ICI) that interferes with the signal between immunity and tumor and adoptive cell therapy that allows immune cells to attack tumor cells (e.g., CAR-T, TILs, etc.). In numerous clinical trials, the effectiveness of immunotherapy on tumors depends on the cancer type and the cancer patient's immune status [2]. Peripheral mononuclear cells (PBMCs) containing a variety of cell types such as T- and B- lymphocytes, natural killer cells (NK cells), dendritic cells (DCs), and monocytes actively respond to tumor cells [3]. Though PBMC is a valuable source for monitoring immune-relevant tumor mechanisms and diagnosing tumor status [3], a comprehensive omics analysis in PBMCs from tumor patients has not been performed. Here, we generated a primary dataset suitable for understanding epigenetic regulation circulating immune cells respond to tumors using PBMCs derived from dog mammary gland tumors.

Epigenetic modification is an essential factor that enhances the effectiveness of cancer treatment by immune cells [4]. Recently, clinical trials have been underway on the combination therapy of ICI with epigenetic drugs such as HDAC inhibitors (HDACi), 5-aza-2-deoxycytidine (5-Aza), and decitabine [5]. DNA methylation is a reversible change and a valuable target that can be modulated and quickly detected [6]. Promoter methylation of checkpoints such as CTLA-4, PD-1, and CD28 has been reported to be associated with systemic suppression of immune cells in the tumor microenvironment (TME) [7]. In addition, methylation of peripheral blood immune cells is a strong candidate for diagnosing solid tumors such as head and neck squamous cell carcinoma [8], liver cancer [9], bladder cancer [10], and ovarian cancer [11]. Understanding epigenetic regulation in circulating immune cells provides valuable information to diagnose tumor type, grade, and prognosis and treat tumors with immune remodeling therapy (e.g., CAR-T therapy) [12]. Nevertheless, many studies in human cancer methylome have focused on tumor-infiltrating immune cells and immune checkpoints. Epigenetic information of PBMC has advantages in providing diagnostic, prognostic, and therapeutic information based on easily accessible liquid biopsy modality.

Since epigenetic responses to environmental factors occur actively in dogs as in humans, comparative medical studies using dogs have been conducted on aging, tumor biogenesis, and inflammatory diseases [13]. It has been reported that dogs might be helpful animal models for immunotherapy studies because they are immune-competent, and their tumor biology is similar to that of humans [14]. Indeed, several recent studies have evaluated the cross-reactivity of immunotherapy against human and canine cancers [15].

We identified epigenetic signatures in circulating immune cells of CMT through a genome-wide methylation study of PBMCs in normal, benign tumors and malignant tumors (carcinoma). We found aberrant methylation of immune regulatory genes involved in various immune cell’s proliferation and normal differentiation. This result suggests that immune cell activity is affected by CpG methylation not only in the tumor microenvironment but also in peripheral blood. Furthermore, we modeled a two-step classifier that can distinguish benign and malignant tumors from normal through machine learning (ML) algorithms using the PBMC methylome datasets.

## Results

### Profiling differential methylation of peripheral blood mononuclear cells in canine mammary gland tumor

**Fig. 1:**
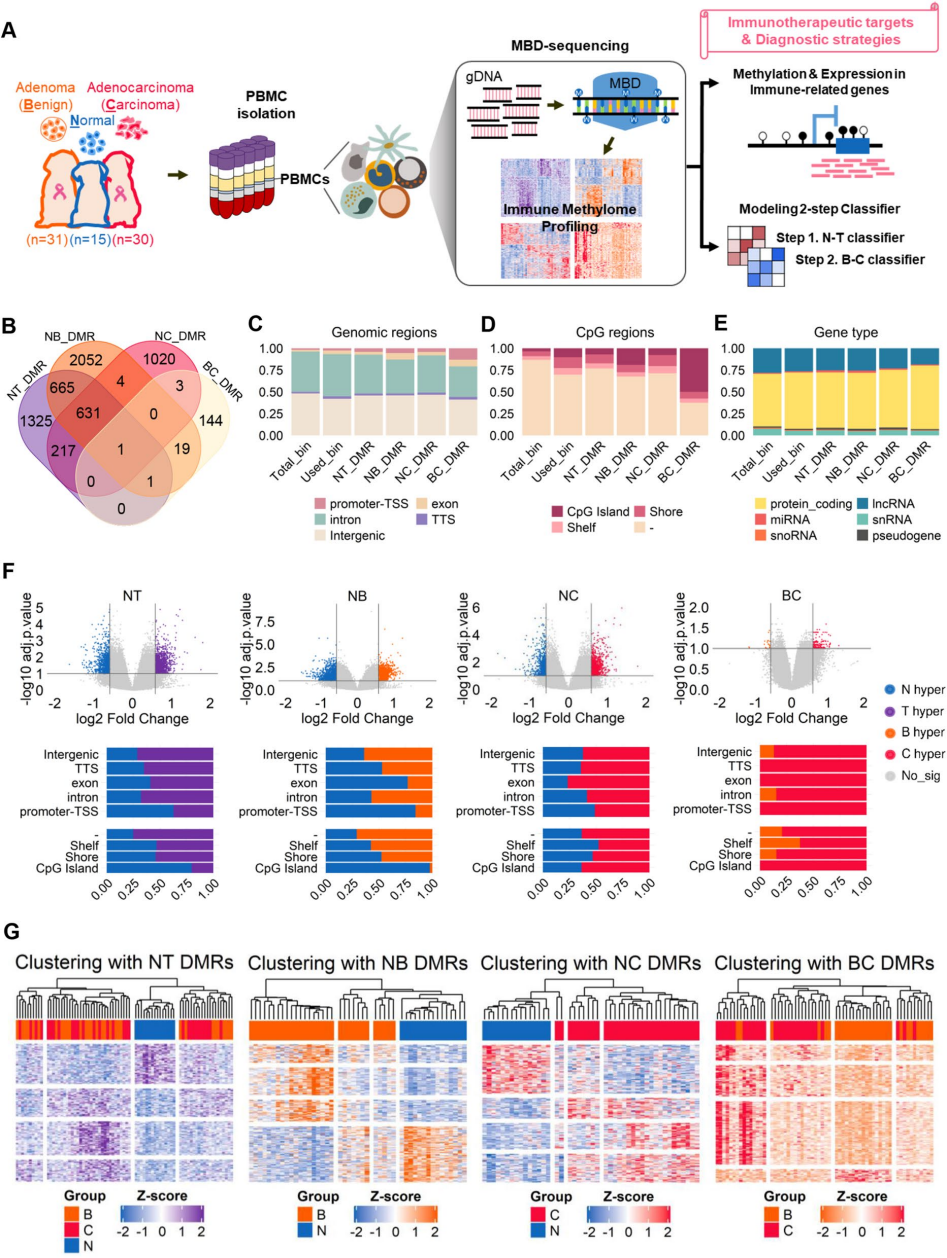
Pair-wise comparison for genome-wide PBMC methylome datasets from benign, carcinoma, and normal dogs. **A** Synopsis of genome-wide PBMC methylome study. **B** A Venn diagram shows the number of common and unique DMRs identified in each comparison (FDR-adjusted *p*-value < 0.1 and log_2_FC ≥  ± 0.585). **C**-**E** The distributions of genomic features in Total bins, Bins_used, and each DMR to see pronounced regions. ‘Bins_used’ regarded signal peaks used for DMR analysis, excluding noise bins (both low signal bins and zero CpG bins) from ‘Total bins’. **F** Volcano plots and 100%-scaled stacked bar plots with the frequency and genomic profile of hypo- and hyper- methylated bins. The x-axis is the ‘log_2_ methylation fold change’, and the y-axis means the statistical significance. Hypermethylated in ‘N’ is expressed as blue, ‘T’ as purple, ‘B’ as orange, and ‘C’ as red. **G** Heatmap Clustering of ‘N and T with NT_DMR (2840 DMRs)’, ‘N and B with NB_DMR (3373 DMRs)’, ‘N and C with NC_DMR (1876 DMRs)’, ‘B and C with BC DMRs (168 DMRs)’. The clustering distance between samples (columns) followed Pearson’s correlation, and the ‘complete’ method was used

We first made genome-wide differential methylation profiles of PBMCs in CMT. To evaluate the genome-wide effects of mammary tumors on PBMC DNA methylation, PBMCs were collected from 15 healthy dogs (Normal; N), 31 dogs with mammary adenoma (Benign; B), and 30 dogs with mammary carcinoma (Carcinoma; C) (Fig. 1A). The donor’s information is listed in Table S1. The healthy samples consist of six dog breeds, aged 1 to 12, and 13 females, including eight spayed females and two neutered males. Patient specimens comprise 16 dog breeds aged 5 to 16 and six significant subtypes of canine mammary tumors (ductal, simple, complex, mixed, inflammatory, and comedo). All patient dogs were females or spayed females.

Global CpG methylomes have enriched and analyzed by methyl-CpG-binding domain sequencing (MBD-seq) that has high coverage in highly methylated CpG and CpG-rich regions (Fig. 1A). The quality check for NGS data has also been performed (Table S2). Sequencing reads more than 5X depth (considered as signal peaks) show about 50% CpG coverage, indicating that the MBD-seq data was successfully produced and informative (Figure S1A). The R Bioconductor MEDIPS (v.1.46.0) [16] was mainly employed to calculate methylation levels and identify differentially methylated regions (DMRs) (Figure S1B). DMRs were further subjected to ML for modeling an immune classifier for CMT. Of the total, 4,655,287 bins (referred to as ‘Total bins’ in Fig. 1C-E) were generated at 500 bp size, and 1,220,164 bins (referred to as ‘Bins_used’ in Fig. 1C-E) with reading counts of 25 or more were used for further analysis.

Together with pair-wise comparisons (Normal vs. Benign (NB), Normal vs. Carcinoma (NC), and Benign vs. Carcinoma (BC)), we also compared Normal vs. Tumor (NT), in which tumor includes benign and carcinoma. From each comparison, 2840, 3373, 1876, and 168 DMRs were identified with significance (log_2_FC ≥  ± 0.585 (|Fold Change|≥ 1.5), adjusted *p*-value (FDR) < 0.1) for NT, NB, NC, BC, respectively (Fig. 1B). The statistics and genomic features of each DMR group are listed in Table S3-S6 for NT_DMR, NB_DMR, NC_DMR, and BC_DMR, respectively. Interestingly, the NB comparison shows the highest number of DMRs, followed by NT. As expected, NT comparison shares more than half of DMRs (1514) with NB and NC comparisons. Of note, DMRs from NB and NC comparisons share 636 DMRs and methylation directions (that is, B-hyper = C-hyper, B-hypo = C-hypo), indicating the methylation status of immune cells against tumors are similar in benign and carcinoma (Figure S2). Most of all, we focused if DMR profiles of PBMC can distinguish corresponding tumor types (benign or carcinoma) as well as Normal. However, only a small number of DMRs were identified from BC, and most BC_DMRs were unique across all DMRs, indicating that they are not explicitly associated with tumor states.

The uniqueness of BC_DMRs was shown in the genomic and CpG regional distribution and gene types linked to DMRs (Fig. 1C-E). Total bins consist of five genomic regions. Compared with the ‘Total bins’, the intron region was increased when the intergenic region was decreased in the ‘Bins used’. Moreover, more numbers of the CpG island, Shore, and Shelf regions were enriched in the ‘Bins used’ compared to the ‘Total bins’. Interestingly, BC_DMRs were enriched in the promoter and exon regions and the CpG island regions, which are more associated with the protein-coding region.

We then analyzed the direction of DMRs using volcano plots and 100% stacked bar charts in eight genomic regions (Fig. 1F). Overall, methylation increased in tumors compared to Normal. In BC, the Carcinoma group was more methylated than the Benign group. Regionally, changes in methylation status were highly dynamic according to the comparison group. In the NB comparison, there were more hypomethylated DMRs in CpG islands, promoter, and exon compared to other regions. Although these characteristics were similarly shown in the NT comparison, hypermethylated DMRs are prominent across all eight regions in the NC comparison.

Nevertheless, exon, promoter, and CpG island regions were highly hypomethylated in the BC comparison. Most of BC_DMRs, indeed, were hypermethylated in carcinoma. It is an essential feature because hypermethylation of certain groups of genes and DMRs might be a cancer-specific signature.

We then tested if DMRs separate each comparison group. The pair-wise hierarchical clustering separated the Normal group from the Benign, Carcinoma, and Tumors groups (Fig. 1G, Figure S3A-B). However, the Benign and Carcinoma groups were not entirely separated from each other, suggesting a new clustering algorithm for PBMC methylome classification for these group differentiation. The PBMC samples used in this study were obtained from dogs with diverse characteristics, including age, gender (neutered or not), tumor subtype, hospital where the blood was collected, and tumor features, among others. To investigate the potential effects of these variables, we performed hierarchical clustering using the NT_DMRs that we identified, to examine their influence (Figure S3C). Our results show that the clustering of normal PBMC and tumor PBMC samples using NT_DMRs was not influenced by the diverse variables between the samples.

### Differential methylation accompanies changes in immune cell populations and proliferation in malignant tumor patients

Several studies have investigated the methylation patterns of blood immune cells, limited to specific target genes and not on a genome-wide scale [17–20]. Since PBMC is a mixture of a wide variety of immune cells, there is a limit to the regulation or role of various immune cells. To this end, single-cell bisulfite sequencing technology has been attempted, but several limitations exist in diagnosing cancer or defining the immune status. We analyzed the whole genome-wide methylation profile obtained from bulk PBMC samples and attempted to confirm various immune status changes in different tumors.

**Fig. 2:**
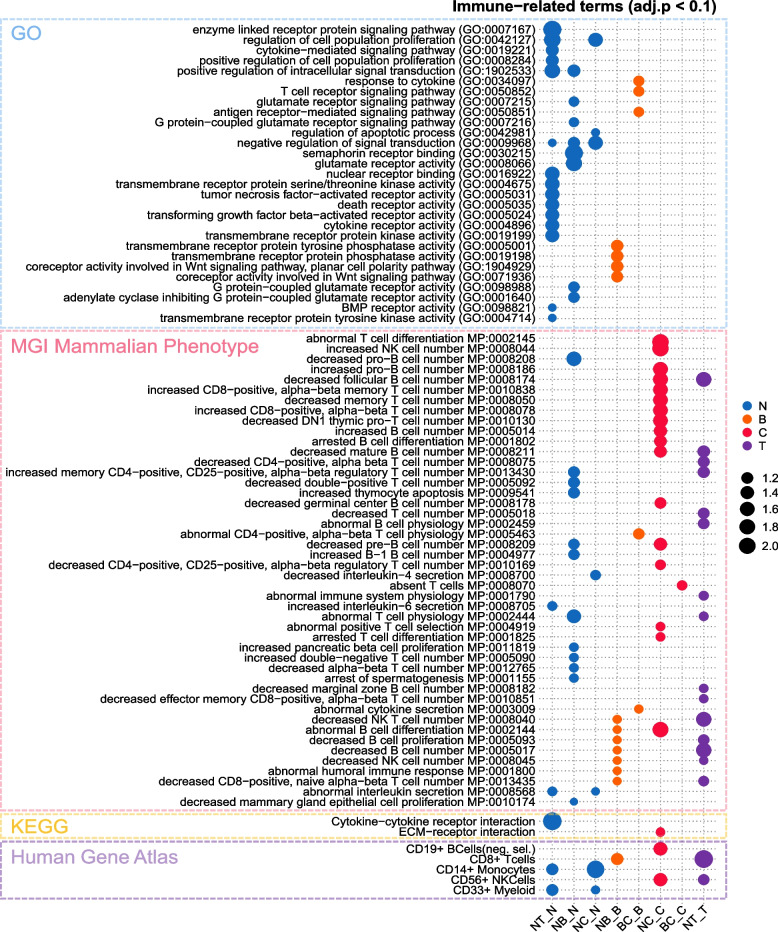
Gene enrichment analysis for DMGs shows differential immune signatures between tumor and normal PBMCs. Immune-related terms significantly enriched in the Gene Ontology (blue box), the MGI Mammalian Phenotype (pink box), the KEGG pathway (yellow box), and the Human Gene Atlas (purple box) are shown. The color of dots means which group is hypermethylated (‘N-hyper’ is expressed as blue, ‘T-hyper’ as purple, ‘B-hyper’ as orange, and ‘C-hyper’ as red. The size of the dots indicates the statistical importance (according to -log10 adjusted *p*-value). The table corresponding to this figure shows the genes included in each term, which is in Table S7

We defined DMGs using DMRs existing in promotor, exon, intron, and TTS and performed gene set enforcement analysis (Fig. 2, Figure S4, and Table S7). Figure 2 shows that the immunocyte-related terms are significantly enriched in Gene Ontology (GO), Mammalian Phenotype Ontology in Mouse Genome Informatics (MGI), and Human Gene Atlas (HGA) databases [21–23]. In all comparative groups, genes involved in signal pathways directly related to cell activity, receptor activity, and cytokine modulation are hypomethylated in tumors (both benign and carcinoma), whereas there is no significant term or pathway found in hypermethylated in carcinoma (Part of ‘GO’ and ‘KEGG’ in Fig. 2).

The MGI and HGA databases, which focus on the function of immune cells, provide clues to infer the immune status in the blood (Part of ‘MGI Mammalian Phenotype’ and ‘Human Gene Atlas’ in Fig. 2). Comparing the normal with the overall tumor, the terms associated with the increase or abnormal function of T-cells, B-cells, and NK cells were high. The comparison between normal and cancer showed that the gene group with higher methylation in cancer PBMC was involved in the increasing or decreasing of B-cells or T-cells. Among T-cell types, the genes associated with the increase in CD8 + T-cells were most highly associated. On the other hand, compared with benign and normal the highly methylated genes in the benign group showed abnormalities in NK and B-cells. The primary immune cell types responding to benign and carcinoma differ. As for the DMR of BC comparison, there was no significant difference in the gene enrichment analysis, as the number was minimal, as shown in Fig. 1B. Through the PBMC DMRs associated with immune responses to tumors, it is expected to find methylation biomarkers that can distinguish the presence or absence of tumors and the malignancy of tumors.

### Immune cell markers functionally involved in cell proliferation and activation of B, T, and NK cells are hypermethylated in tumor PBMCs

**Fig. 3:**
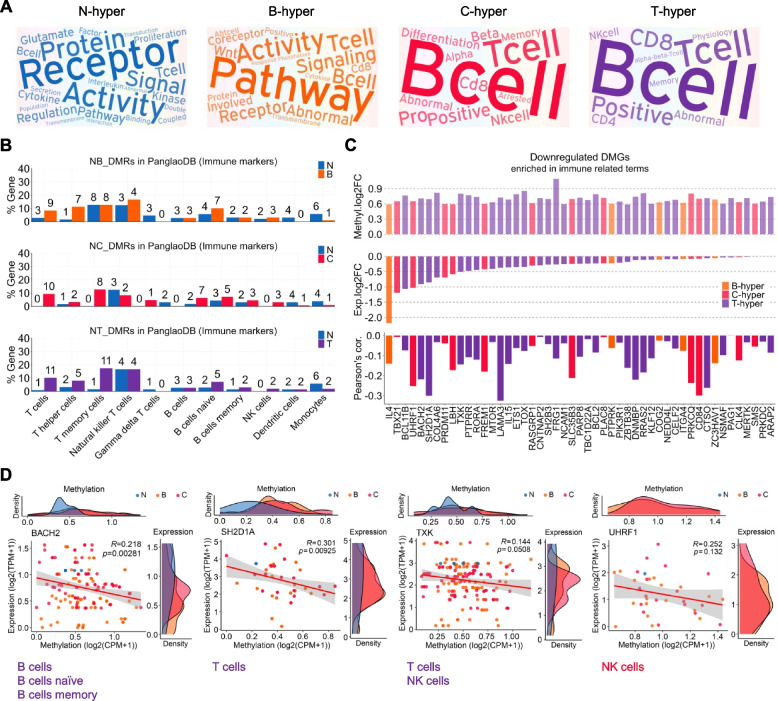
Immune cell markers involved in normal proliferation and activation of B-cells, T-cells, and NK cells are hypermethylated in tumor PBMCs. **A** Text clouds intuitively show the frequency of words enriched in immune-related terms. The color of the text indicates which group is hypermethylated (‘N-hyper’ is expressed as blue, ‘T-hyper’ as purple, ‘B-hyper’ as orange, and ‘C-hyper’ as red). The meaning of the four colors (blue, purple, orange, and red) was applied equally to the following graphs in this figure. **B** The number of hypermethylated genes included in immune cell type markers is expressed as a percentage (%) of total genes in the corresponding cell type. The number of matched genes is displayed on the top of each bar. The list of marker genes for 11 types of immune cells was downloaded from Panglao DB. **C** Among genes enriched in significant immune-associated terms, hypermethylated DMGs that reversely correlate with expression are shown. The y-axis of the bar graph on top means log_2_ fold change of methylation values, and that of the middle one means log_2_ fold change calculated using TPM values derived from RNA-seq. The y-axis of the bottom one shows the degree of inverse correlation between methylation and expression by Pearson’s correlation. Hypermethylated genes included in Panglao DB and its genomic features are listed in Table S8. **D** The scatter plots with linear regression (red line) in 4 representative genes among 49 genes listed in (**C**). The Pearson correlation coefficient, expression (fold-change), and methylation (fold-change) for every immune-related DMGs are also described in Table S9

Through gene enrichment analysis (Fig. 2), we could expect that methylation of immune cells in tumor patient dogs is involved in the population or activity of specific cell types. The gene enrichment analysis mapped the highest terms. Using text mining for meaningful GO terms in adj. *p* < 0.1, words containing ‘receptor’, ‘signal’, ‘activity’, ‘pathway’, ‘T cell’, and ‘B cell’ were prominent in all comparisons (Fig. 3A). These enrichments suggest that hypermethylation occurs in immune cells responding to tumors and is involved in signal transduction of immune cells. To confirm whether the methylation change in PBMC is due to the alteration of immune cell populations and or the cell activity, we investigated the DMR distribution on the immune cell type marker genes in PanglaoDB (Fig. 3B). DMGs included in 11 types of immune cell markers are listed in Table S8. First, NB_DMRs was found increasingly on the marker genes of naive B-cells, T-cells, and T helper (Th) cells. Instead, NC_DMRs were found more in B-cells, NK cells, and many subtypes of T-cells. NT_DMRs were found more in naive B-cells, NK cells, and T, Th, and T memory cells, combined with NB and NC. On the contrary, it is of note that myeloid lineage cells, such as monocytes are decreased in tumors.

We then identified the most influenced genes by altered methylation among the cell type markers. Figure 3C shows the cell type marker genes highly enriched in the immune-related GO terms considering the gene expression levels. IL4 was most frequently altered in the GO terms, and the expression decreased significantly. The list of genes, including TBX21, BCL11B, UHRF1, BACH2, SH2D1A, COL4A6, PRDM11, LBH, and TXK, showed tumor-associated hypermethylation and a significant negative correlation to gene expression. The fold-change of methylation and expression and correlation coefficient calculated for every immune-related DMGs are also listed with corresponding DMRs’ genomic features in Table S9. We integrated RNA-seq data to show an association between methylation and gene expression in representative marker genes (Fig. 3D). Among them, BACH2, a B-cell marker; SH2D1A, a T-cell marker; TXK, an NK cell marker; and UHRF1, known to be related to NK cell number, showed a significant negative correlation between the RNA expression and overall gene methylation. These results showed that the well-enriched immune cell markers in the genome-wide methylation changes are closely linked to gene expression and affect overall tumor immune cell activity.

### Bisulfite-sequencing validated the tumor-associated differential methylation in immune cell marker genes

**Fig. 4:**
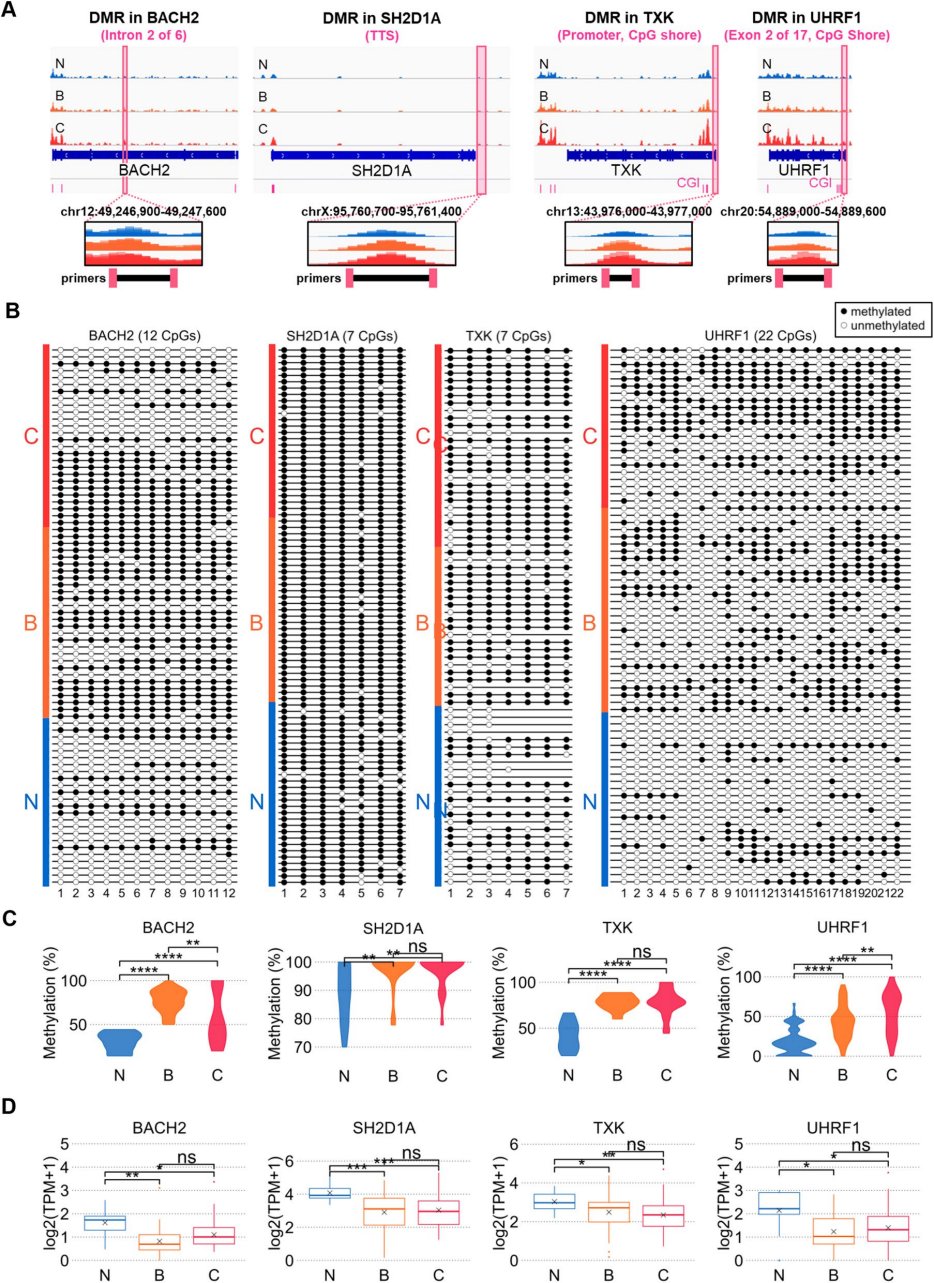
Targeted CpG methylation and expression analysis in representative hypermethylated genes related to immune cell activation. **A** Methylation peaks in four interesting gene regions are shown. Pink dumbbells also express the loci where primers have been designed. The DMR in the BACH2 gene is located in the second intron of 6 introns, the DMR in the SH2D1A gene is located in TTS, DMR in TXK is located CpG shore promoter, and the DMR in UHRF1 is located in the second exon of 17 exons overlapped with CpG shore. **B** The methylation validation for 12 CpGs in BACH2 DMR, 7 CpGs in SH2D1A and TXK DMR, and 22 CpGs in UHRF1 DMR by performing targeted bisulfite sequencing using primers listed in Table S10. Methylated CGs are indicated by black circles, and unmethylated CGs are expressed by empty (white) circles. **C** Violin plots show the distribution of methylated CG (%) between groups. The total percentages of methylated CG were calculated as ‘(The number of methylated CG / The number of total CG in the amplified region) * 100 (%)’ in each CG for every sample. **D** In contrast to Violin plots in (**C**), Box plots show the expression levels are significantly down-regulated in Benign and Carcinoma PBMCs versus Normal PBMCs. The y-axis means the log_2_-transformed (TPM + 1) quantified using RNA-seq

We showed that hypermethylation and gene expression of cell-specific gene markers are inversely correlated with integration analysis of MBD-seq and RNA-seq (Fig. 3C & D). Representative DMRs, which have a reverse correlation with the gene expression, verified the methylation status in vitro by the targeted bisulfite-sequencing (BS-seq). BACH2, an active marker gene of B cells, has hypermethylated DMRs consisting of 11 CpGs on the second intron out of six introns in tumors (benign and carcinoma). The SH2D1A gene, a T-cell activity-related marker, has a hypermethylated DMR consisting of seven CpGs in the TTS region in tumors. A representative carcinoma-related hypermethylated DMR was identified from the CpG shore location, consisting of nine CpG promotor-TSS regions of the TXK gene. A DMR harboring 22 CpGs, which were hypermethylated in carcinoma, was identified from the CpG shore region located in the second exon among 17 exons of the UHRF1 gene (Fig. 4A). The four pairs of primers targeting the flanking regions of DMRs used for BS-seq are described in Table S10.

Overall, the DMRs from the MBD-seq analysis were confirmed in the targeted BS-seq. However, the methylation frequency varied from each CpG (Fig. 4B). The targeted DMR of BACH2 was the most hypermethylated in benign, followed by carcinoma. DMR on the UHRF1 was most highly methylated in carcinoma, followed by benign. The methylation levels of TXK were similarly high in benign and carcinoma. In the case of SH2D1A gene sites, only the 5th CpG site was a differentially methylated CpG in tumors. This can still be sufficiently meaningful because studies have reported that even the presence or absence of methylation of a single CpG can affect transcription level and cell type specificity [24]. Figure 4C shows the distribution of methylation percentage across samples calculated as the number of methylated CpGs/total number of clones * 100 (%). The RNA-sequencing results performed on PBMCs of CMTs and normal dogs showed a significant decrease in the expression of these four genes (Fig. 4D). When compared between the methylation (Fig. 4C) and gene expression (Fig. 4D), overall methylation levels on the targeted regions by BS-seq were significantly opposite to RNA expression data. Our targeted BS-seq results confirmed that the high-throughput sequencing analysis after methylated CpG enrichment showed relevant genome-wide methylation status in PBMC samples. It then identified DMRs that may directly link to gene expressions that have crucial roles in cell activity and populations in PBMCs. Validation of MBD-seq results through BS-seq increases the likelihood that they can be developed for clinical tumor diagnosis.

### Computational modeling of a PBMC methylome-based two-step classifier distinguishes benign and malignant as well as healthy conditions

**Fig. 5:**
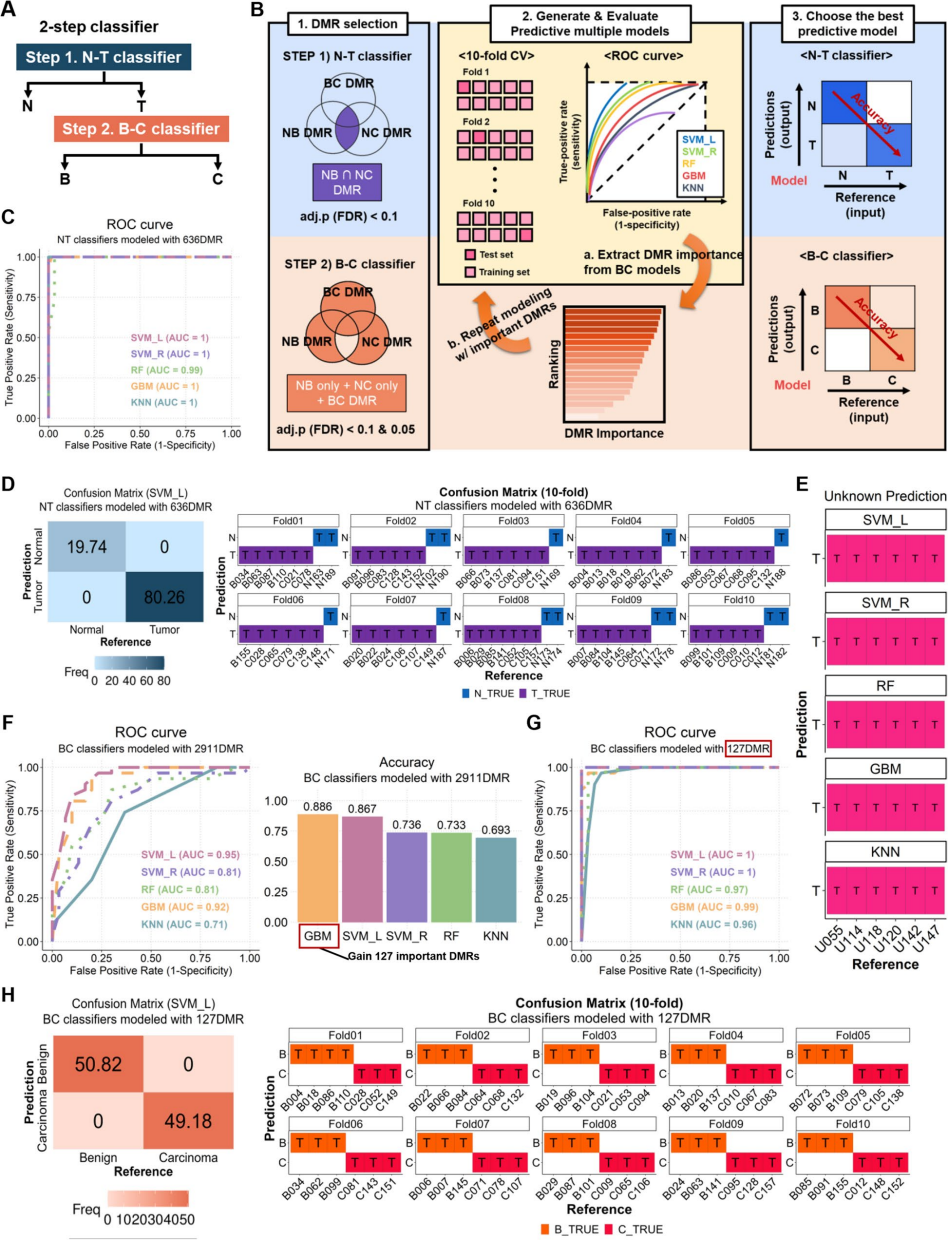
A machine learning-based diagnostic two-step classifier discriminating tumor from normal PBMCs followed by carcinoma from benign PBMCs. **A** The concept of a two-step classifier for precisely distinguishing three groups (Normal, Benign, and Carcinoma). **B** Schematic diagram of the diagnostic methylome-based classifier modeling. To generate the best predictive model, tenfold cross-validation with multiple ML algorithms were employed, and then the performance of each model was evaluated. **C** The ROC curves of the NT classifiers were established by SVM_L, SVM_R, RF, GBM, KNN, and logistic regression. AUC values are shown in the right-bottom area under the curves. **D** Heatmap of the confusion matrix (left) for tumor detection by the SVM_L-based NT classifier, which has the best AUC value (AUC = 1) and accuracy (Accuracy = 1). The confusion matrix for tenfold cross-validation (right) shows the prediction results for seven to nine test samples in each fold. **E** Validation of the predictive performance in multiple NT classifiers. PBMC MBD-seq data from six dogs with CMT were used as the validation set. Except for the logistic classifier, which incorrectly predicted three out of six, the SVM_L, SVM_R, RF, GBM, and KNN classifiers predict tumors. **F** The ROC curves (left) for the BC classifier modeled with 2911 DMRs containing ‘BC_DMR’ and DMRs identified ‘only in NB_DMR’ or ‘only in NC_DMR’. BC classifiers show lower AUC values compared to NT classifiers. The bar graph (right) exhibits the highest accuracy in GBM. 127 DMRs extracted by GBM-based feature importance are used for BC classifier re-modeling. This iterative process is illustrated in the center of (**B**). **G** The ROC curves of re-modeled BC classifiers using 127 DMRs, which show enhanced performance compared to previous BC classifiers. **H** The improved performance was confirmed via both a heatmap of the confusion matrix (left) and the tenfold confusion matrix (right) for the final BC classifier (SVM_L) generated using 127 DMRs

Methylome-based classification is a potential diagnostic method that reflects the stage or subtype of tumors. Previous studies have reported the usefulness of tissue methylation-based classifiers in diagnosing CNS tumors [25], bone sarcoma [26], and renal cell carcinoma [27]. Recently, a model using DNA methylation for discriminating cancer from para-cancerous tissue has been developed [28]. To develop a liquid biopsy-based diagnosis, we attempted to establish a model for diagnosing mammary gland tumors using our genome-wide methylome data. Our results thus far showed immune methylome dynamics between normal and tumor PBMCs. However, it was difficult to define specific DMRs or functional terms that differentiate between benign and malignant tumors by PBMC DMRs. For efficient modeling, we devised a method to classify normal and tumor in step 1 (NT classifier), then classify benign and carcinoma in step 2 (BC classifier) and named it a two-step classifier (Fig. 5A). The process for modeling and performance evaluation is depicted in Fig. 5B.

First, NT classifier modeling was performed using 636 common DMRs with FDR-adjusted *p*-value < 0.1 and log_2_FC ≥  ± 0.585 in NB DMR and NC DMR (Fig. 5C-E). To overcome the problem that arising from the limited number of samples, tenfold cross-validation (tenfold CV) was applied. The classifiers were modeled with five ML algorithms (Support Vector Machine with the linear kernel (SVM_L) or the radial kernel (SVM_R), Random Forest (RF), K-Nearest Neighbor (KNN), Gradient Boosting Machines (GBM), and Logistic Regression), and the performance of each was evaluated with the ROC curve (Fig. 5C). NT classifier shows strong performance with AUC = 1 in SVM_L, SVM_R, GBM, and KNN models except for RF (AUC = 0.99) and logistic regression (AUC = 0.7). In both the representative SVM_L confusion matrix and the tenfold validation result, it is confirmed that benign and carcinoma are classified as T (Tumor) and normal as N (Normal) (Fig. 5D). The accuracy of each model is shown in Figure S5A. The high accuracy and AUC values of NT classifiers remind us that the PBMC methylome profile in tumors is entirely different from normal. To evaluate the predictive ability of the NT classifiers, PBMC MBD-seq data from 6 dogs with mammary gland tumors that were not used for methylome profiling due to uncertain diagnosis were validated in the five NT classifier models (Fig. 5E, the information of 6 unknown donors is listed in Table S11). All of the five NT classifiers exactly diagnosed total six PBMC samples derived from unknown MGT dogs as T (Tumor).

Next, a BC classifier was developed using significant DMRs with FDR-adjusted *p*-value < 0.1 and log_2_FC ≥  ± 0.585 only in NB_DMR and NC_DMR and additional BC_DMR (NB only + NC only + BC DMR = total of 4,122 DMRs). Since the original BC_DMRs with FDR-adjusted *p*-value < 0.1 failed to cluster benign and carcinoma (Fig. 1G), the same modeling process was performed using 2,911 DMRs with FDR-adjusted *p*-value < 0.05 (Fig. 5F-H, Figure S5D-E). The BC classifier trained with the 2,911 DMRs showed the highest performance when using SVM_L (AUC = 0.95), followed by GBM (AUC = 0.92). However, the accuracy of SVM_L and GBM was 0.867 and 0.886, respectively, lower than that of the NT classifier (Fig. 5F). The accuracy was about 0.85, which was inferior to that of the NT classifier (Figure S5B). To improve the performance of the BC classifier, the modeling process was repeated one more time with DMRs of high importance in the initially selected model to increase the discrimination between benign and carcinoma (depicted in Fig. 5B). The performance of the models was measured using 127 DMRs, which showed high relative importance in GBM and the highest accuracy in the primary BC classifier (see the bar graph in Fig. 5F). The degree of feature importance and genomic features of 127 DMRs are shown in Table S12. It shows improved accuracy and performance than the first-order classifier using 2,911 DMRs (Fig. 5G-H, Figure S5B-C). As mentioned above, a parallel analysis was also executed with 4,122 DMRs with an FDR-adjusted *p*-value < 0.1 (Figure S5D-E). The performance of the primary classifier was similar to that using 2,911 DMRs. However, the remodeled classifier using 102 DMRs of high importance in GBM showed slightly lower accuracy than the previous classifier in the confusion matrix of Supplementary Fig. 6E. Both BC classifiers developed with important DMRs have the highest AUC values and accuracy in the SVM_L model. BC_DMR did not differentiate between benign and carcinoma (Fig. 1G). We performed PCA analysis to evaluate whether the DMRs selected for the classifier modeling discriminate between benign and carcinoma (Figure S6). DMRs with higher importance divide the two groups better, indicating that the GBM-based feature importance is relevant. We designed an optimal two-step classifier by utilizing various ML methods and comparing the performance of predictive models. Our result suggests a new diagnostic strategy using the PBMC methylome that can differentiate between normal, benign, and malignant tumors by liquid biopsy.

We constructed a machine-learning-based classifier for diagnosing malignant tumors using PBMC Methylome. To ensure reliability of methylome classifiers, we also modeled the two-step classifier using transcriptome data with the same parameters (Figure S7). The NT classifier demonstrated the highest performance, with an AUC of 0.99 in the GBM model, followed by SVM_R with an AUC of 0.97, which showed a similar performance to the methylome-based NT classifier. The initial BC classifier showed the highest predictive performance, with an AUC of 0.66 in SVM_R. To improve the diagnostic accuracy, we conducted secondary modeling of the BC classifier using features with high relative importance, similar to what was done in the methylome-based BC classifier. However, despite these efforts, the re-modeled BC classifier did not demonstrate improved performance, as indicated by an AUC of only 0.68 in SVM_L. This suggests that methylome data provides more informative and suitable data for discriminating malignant tumors using PBMCs compared to transcriptome data.

## Discussion

This study provides a better understanding of genome-wide epigenomic alteration, presenting a new platform for diagnosing malignant tumors from both normal and benign tumors based on liquid biopsy and DNA methylation sequencing. In several studies, blood-based DNA methylation has been profiled to develop a robust diagnostic marker for cancer. The blood-based methylation studies are broadly divided into investigating global DNA methylation [29] and gene-specific targeted DNA methylation [18]. In addition, according to the source of DNA, these studies mainly targeted circulating tumor cells (CTCs) and cell-free DNA in serum or plasma [30]. In the meantime, methylation of repetitive elements was generally investigated as surrogates for genome-wide DNA methylation measurement [31]. There have been consistent attempts to diagnose breast cancer (BC) patients using peripheral blood. BC is the most common malignant tumor in women worldwide. The prognosis of BC mainly depends on early detection; to this day, it primarily relies on mammography. CA15-3 or CA27.29 [32], approved by the FDA as blood-based protein biomarkers for BC, are recommended only for monitoring disease recurrence and therapeutic efficacy rather than diagnosis. Recently, several studies have reported genome-wide blood DNA hypomethylation in BC patients [33]. Hypermethylation of the BRCA1 gene in the blood cells and the RASSF1A gene in cfDNA has been reported in BC patients [19]. On the contrary, some studies have also reported an association between low methylation of immune cells and increased BC risk. Thus, the evidence still needs to be more conclusive. It suggests that reliable epigenomic information based on PBMC for diagnosing BC and predicting therapeutic efficacy are needed to be studied in detail and cross-species approaches. Therefore, we performed genome-wide methylome analysis in the canine PBMC with CMT as an alternative approach for BC.

Recently, many studies have revealed that methylation, not only in the promoters but also in gene body regions such as exon, intron, and TTS regulates transcription [34]. For this reason, methylation profiling on a genome-wide scale has been steadily attempted to confirm the distribution of DMR at various locations targeting only specific genes. Since the CpG region is also an area in which epigenetic dynamics are actively occurring due to the recovery of methyltransferase and histone modifiers, it is also imperative to understand the DMR distribution from CpG islands and their surroundings (shore and shelf regions). Although CpG islands account for only 4 to 5% of the genome, approximately 70% of promoters are associated with CpG islands affecting directly annotated gene regulation [35]. Recently, the ± 2 kb region on both sides of CpG islands (called ‘CpG shore’) has been reported to be associated with cell type specificity and highly correlated with gene expression [36]. Therefore, these methylation changes in various regions of the blood cell genome in cancer patient dogs can affect gene expressions in cancer immunity. In this study, we observed the increased methylation of CpG shore in TXK and UHRF1 strongly anti-correlated with gene expression. Although hypermethylation of CpG islands was prominent in PBMCs with carcinoma, DMRs in the CpG shore region showed a significant inverse correlation with gene expression. However, since PBMC methylome has more variables depending on the cell type and composition, our study has limitations in elucidating the epigenetic regulation dependent on the CpG region.

PBMC has been used in various blood target studies conducted in clinical use. However, a recent study raised the question of whether PBMC transcriptome can reflect the actual state of the blood [37]. It is because PBMC contains a wide range of cells that may vary in number from patient to patient rather than a homogeneous cell population. Fortunately, projects such as the ENCODE Project and Roadmap Epigenomics have shown widespread commonality in these different cell types of transcription, but there are still distinct differences among cell types. It means that a significant difference may not be detected in PBMC if different cell types are oppositely methylated comparing two groups of DMRs. For instance, if DMRs have high methylation in T-cells but low methylation in other cells, those differences may be offset and undetected. To overcome this limitation, trials to understand PBMC data in single-cell levels via computational deconvolution or perform single-cell epigenomics are required; however, studies on PBMC methylation in single-cell resolution have not been widely conducted yet.

T-cells are vital immune mediators, differentiating into multiple subtypes in response to cancer. For this reason, T-cells have been regarded as valuable immunotherapeutic targets, and studies on tumor-infiltrating lymphocytes (TILs), immune checkpoints, chimeric antigen receptor-engineered T cells (CAR-T), and TCR-engineered T cells (TCR-T) have been reported [38]. T-cells are programmed to attack tumors by recognizing tumor-derived antigens and secreting anti-tumorigenic cytokines [39]. Our gene enrichment analysis confirmed the aberrant methylation of genes associated with abnormal T cell differentiation as well as decreased CD8 + T cell number in cancer PBMCs. This suggests that DNA methylation is an essential key to improving the effectiveness of cancer immunotherapy in ameliorating the systemic disorder of T cells in tumors.

Hypomethylated promoters with the upregulated gene expressions of PD-1, CTLA4, and TIM3 are reported in primary breast cancer tissues [17], and CTLA4 and TIGIT promoters in colorectal cancer tissues [40]. Unlike these epigenetic characteristics shown in tumor tissues, it has been reported that methylation and expression patterns of immune checkpoints are different in peripheral blood immune cells [18]. This indicates that genome-wide scale studies on the methylome of circulating immune cells are essential to depict T-cell dysfunction and abnormal differentiation. Our PBMC methylome profiling of canine mammary tumors showed that genes involved in the differentiation and proliferation of T-cells, B-cells, and NK cells are abnormally hypermethylated. We observed increased methylation and downregulation of four representative genes (BACH2, SH2D1A, TXK, and UHRF1). BACH2 and SH2D1A are closely related to the proliferation and activation of T cells and B cells [41, 42]. TXK is involved in the significant kinase signaling pathway regulating TCR signaling along with Tec family kinases ltk and Rlk [43]. The evidence that UHRF1 is directly related to immune cell activity is insufficient. A study described that tumor-derived exosomal circulating UHRF1 promotes NK cell exhaustion in hepatocellular carcinoma [44]. Since UHRF1 is known to interact with methyltransferase to regulate the expression of other genes, it is required to study further whether methylation and expression of UHRF1 in cancer immunity are related to T-cell dysfunction.

Overall, our study highlights the unexpected epigenetic regulatory layer in silencing the activation of select circulating immune cells via hypermethylation which further associates tumor malignant states.

This hints at the possibility that the mechanism of immune exhaustion in the circulation differs from that in local TMEs. This is probably because circulating immune cells are less educated by tumors. Immune exhaustion in the peripheral blood can be explained through the expression of cell type-specific genes or kinetic pathways involved in cell activation rather than immune checkpoints. Although these assumptions require experimental validations, we exploited these genome-wide PBMC methylome profiles to develop a classification framework for biomarker discovery.

## Conclusions

In this study, we first profiled the genome-wide methylome in PBMCs of canine mammary gland tumors using MBD-seq. By comparing the PBMC methylomes in normal, benign, and malignant tumors, we found that benign and cancer PBMCs had distinct methylome profiles from those of normal PBMCs. We identified four hypermethylated genes (BACH2, SH2D1A, TXK, and UHRF1) involved in T-, B-, and NK cell activity and inversely correlated with gene expression by RNA-seq. Furthermore, we developed the PBMC methylome-based diagnostic classifier that distinguishes between normal and tumor and benign and malignant tumors through ML technology. Our study provides an understanding of comprehensive epigenetic regulation of circulating immune cells in response to the tumor environment. Furthermore, we present a new paradigm for diagnosing benign and malignant tumors based on liquid biopsy PBMC DNA methylation. Our results also deliver valuable information on immune cell DNA methylation for immunotherapy in terms of therapeutic decision-making and prediction of therapeutic efficacy.

## Methods

### Clinical samples

The protocol was approved by the Institutional Review Board (IRB) of Seoul National University (IACUC SNU-170602–1) and the Institutional Animal Care and Use Committee (IACUC). Blood samples from healthy dogs and dogs with clinically diagnosed mammary tumors were collected in EDTA tubes. For PBMC isolation, 1-2 ml of blood was carefully transferred to a 2X volume of Ficoll-Paque PLUS (GE Healthcare, 17144002) and centrifuge at 400 g. After washing with phosphate-buffered saline (PBS), obtained PBMCs were fresh-frozen for storage or used for following MBD sequencing, target BS sequencing, and total RNA sequencing. Clinical information for normal and mammary tumor dogs is presented in Table S1.

### Methyl-binding domain (MBD) sequencing

MBD sequencing was performed as previously reported by our group [45]. Briefly, genomic DNA has been isolated from dog-derived PBMCs using the DNeasy DNA Extraction Kit (QIAGEN, 69504). After 3 μg of genomic DNA was sonicated, MBD-biotin was incubated with Dynabeads-streptavidin and bound to 500 ng of dsDNA. MBD-enriched DNA was obtained from 600 and 800 mM elutes which contain highly methylated DNA fragments. MBD-enriched DNA was subjected to library construction and sequenced by Illumina Hiseq 4000 next-generation sequencing platform (Illumina, CA, USA).

### Genome-wide methylome profiling

Quality check, trimming, alignment, and quantitation processes for MBD-seq data were executed as detailed in our previous methylome study [45]. We calculated raw counts ​​for bins (called ‘Bins_used’ in Fig. 1C-E) excluding low signal bins and zero CpG bins using the ‘*MEDIPS.createROIset’* function of MEDIPS R Bioconductor [16]. We performed pairwise DMR analysis for the Bins_used by applying the ‘*MEDIPS.meth*’ function of MEDIPS. We set specific parameters (p.adj = “fdr”, diff.method = “edge R”, minRowSum = 1000, diffnorm = “quantile”), the bins with FDR-adjusted *p*-value < 0.1 and log_2_FC ≥  ± 0.585 (same as fold change upper 1.5) were defined as significant DMRs. Quantile normalized counts and log_2_ transformed CPM values ​​were used for plotting and quantitative analysis. In addition, we counted reads in every 50 bp across the whole genome using the source code of MethylAction (https://github.com/jeffbhasin/methylaction) to generate high-resolution ‘bigwig’ files for visualizing methylation peaks in the Integrative Genome Viewer (IGV v.2.8.0) [46].

### Annotation of methylation peaks

Information on genomic features of CanFam3.1 (v99), a dog reference genome, was obtained in a GTF format from Ensembl Genome Browser (release 104, May 2021). `Promoter-TSS` means extended regions around TSS from -1000 bp to + 100 bp, while `TTS` indicates extended regions around TTS from -100 bp to + 1000 bp. We downloaded the genomic location of CpG islands from the UCSC Genome Browser and named the region extending $\pm$ 2 kb from the CpG island as ‘CpG shore’ and the region extending from $\pm$ 2 kb to $\pm$ 4 kb from the CpG island as ‘CpG shelf’. Total bins, Bins_used, and DMRs were annotated to the prepared genomic information using the ‘*annotatePeaks.pl*’ function provided in HOMER v4.11.1.

### Functional enrichment analysis

We investigated the enriched terms for DMGs using EnrichR (a web server for the comprehensive gene set enrichment analysis: maayanlab.cloud/Enrichr/) [47] to elucidate the function of genes undergoing aberrant methylation. Because most functional terms are derived from human and mouse, we converted dog Ensembl IDs into human orthologous gene symbols using multiple species datasets downloaded from the Ensembl BioMart (Ensembl Genes 104). Finally, we found significant functional terms in various libraries such as Gene Ontology (GO), KEGG pathway (2021), MGI Mammalian Phenotype (Level 4, 2021), and Human Gene Atlas (see Fig. 2). Panglao DB is a web database that shares single-cell RNA sequencing data conducted on human and mouse [48]. We extracted a list of marker genes for 11 immune cell types corresponding to the composition of PBMC included in the immune system from the Panglao DB. This list was used to identify methylation changes in cell marker genes (Fig. 3B).

### Targeted Bisulfite-sequencing (BS-seq)

Targeted BS-seq was performed using genomic DNA from 9 PBMC samples, including PBMCs used for MBD-seq (*n* = 3 in normal (N), benign (B), and carcinoma (C), respectively). We designed bisulfite primers using the Bisulfite Primer Seeker (https://www.zymoresearch.com/pages/bisulfite-primer-seeker). The overall process of targeted BS-seq was conducted as previously described [49]. The primer sequences are listed in Table S10). Subsequently, the sequences were aligned to the reference sequence in the amplified region using MEGA v11.0.11 [50]. The methylation (%) for the whole CpGs in each region was calculated and visualized as violin plots. To compare the methylation levels between different groups each other, the *t*-test was employed.

### Classifier modeling and evaluation

We calculated the log (CPM + 1) values for the entire bins to generate the methylome-based classifiers, while log (TPM + 1) was used for modeling transcriptome-based classifiers. Five ML algorithms; 1) Support vector machine (SVM) with linear kernel, 2) SVM with the radial kernel, 3) Random Forest (RF), 4) Gradient Boosting Machines (GBM), and 5) K-Nearest Neighbor (KNN) were compared to construct an optimal classifier. We estimated the performance of the ML algorithms through the tenfold cross-validation (tenfold CV) to reduce the overfitting of models. In this process, the hyperparameters in each model were selected by default because we chose an ML algorithm to find DMRs that generally classified the groups well using R package caret (v6.0.85) [51]. The two-step classifier consists of an NT classifier that distinguishes tumors from normal and a BC classifier that distinguishes carcinoma from benign tumors using PBMC methylome. Although both classifiers were constructed through the same computational modeling process, there was an additional modeling step based on feature importance to enhance the performance of the BC classifier. The optimal BC classifier was designed with 127 DMRs, which had high feature importance from the GBM classifier with the highest accuracy among the primary models (Table S12). Feature importance was calculated based on nested cross-validation using the R package gbm (v2.1.8). We evaluate multiple classifiers using the prediction accuracy and area under the ROC curve (AUC) using the R package pROC (v1.18.0) [52].

### Statistics

Statistics and statistical tools for each analysis have been described above. The correlation coefficient between DMR methylation and gene expression was calculated by Pearson correlation and regression analysis. Comparison for the expression between The t-test was implemented to compare gene expression between groups. The number of asterisks between the two groups indicates the degree of statistical significance. If there was no statistical difference between the two groups, it was expressed as ‘ns (not significant)’ without an asterisk. We exploited Rex (v3.6.1) [53] and R (v4.0.2) in NGS data quantification, statistical analyses, and classifier modeling.

## Supplementary Information

**Additional file 1:** **Figure S1. **Quality check and processing MBD-seq data. **Figure S2. **Venn diagram for hyper- and hypo-methylated DMRs. **Figure S3. **Unsupervised and supervised clustering between comparison groups. **Figure S4. **Enriched terms ranked in the Top 3 by combined score according to comparison groups. **Figure S5. **Evaluating the accuracy and predictive performance of the two-step classifier. **Figure S6. **PCA analysis using DMRs involved in the BCclassifiers. **Figure S7. **The predictive performance of transcriptome-based two-step classifier.

**Additional file 2:** **Table S1.** The information about dog donors providing blood samples used for MBD-seq. **Table S2.** Sequencing Coverage and Quality Statistics. **Table S3.** The statistics and genomic features of NT_DMR (2840 DMRs). **Table S4.** The statistics and genomic features of NB_DMR (3373 DMRs).**Table S5. **The statistics and genomic features of NC_DMR (1876 DMRs). **Table S6.** The statistics and genomic features of BC_DMR (168 DMRs). **Table S7.** The list of enriched immune-related terms with adj.*p* <0.1. **Table S8.** The list of hypermethylated DMRs in immune cell type markers (Panglao DB). **Table S9. **The list of immune-regulating DMGs inversely correlated with gene expression. **Table S10.** The list of primers designed for targeted BS-sequencing. **Table S11.** The information of unknown dog PBMC donors (used for validation sets of NT classifier). **Table S12.** The list of 127 DMRs which have high feature importance in BC classifier.

## Data Availability

The NGS data for a total of 76 MBD-seq samples have been deposited in the NCBI SRA database under accession numbers SRR21527808-SRR21527889 in BioProject PRJNA879244. Additionally, the transcriptome data comprising a total of 64 RNA-seq samples have been deposited with SRA accession numbers SRR23336423-SRR23336486 in BioProject PRJNA931332. Other raw data supporting this study are supplemented in Supplementary Figures and Tables and provided in this manuscript.

## Abbreviations

BACH2: BTB Domain and CNC Homolog 2
CAR-T: Chimeric antigen receptor T cell
CpG: Cytosine and guanine separated by phosphate
CMT: Canine mammary tumor
DMG: Differentially methylated gene
DMR: Differentially methylated region
MBD-seq: Methyl CpG binding domain sequencing
MGT: Mammary gland tumor
ML: Machine learning
PBMC: Peripheral blood mononuclear cell
PCA: Principal component analysis
ROC: Receiver operating characteristic curve
SH2D1A: SH2 domain containing 1A
TCR: T cell receptor
TIL: Tumor infiltrating lymphocyte
TME: Tumor microenvironment
TSS: Transcription start site
TTS: Transcription termination site
TXK: Tyrosine kinase
UHRF1: Ubiquitin like with PHD and ring finger domains 1
