# Supplementary material for: The landscape of PBMC methylome in canine mammary tumors reveals the epigenetic regulation of immune marker genes and its potential application in predicting tumor malignancy
Source: BMC Genomics. 2023 Jul 18;24:403. doi: 10.1186/s12864-023-09471-6 (PMC10353108; doi:10.1186/s12864-023-09471-6)
Supplement: Supplementary file 1 — Additional file 1: Figure S1. Quality check and processing MBD-seq data. Figure S2. Venn diagram for hyper- and hypo-methylated DMRs. Figure S3. Unsupervised and supervised clustering between comparison groups. Figure S4. Enriched terms ranked in the Top 3 by combined score according to comparison groups. Figure S5. Evaluating the accuracy and predictive performance of the two-step classifier. Figure S6. PCA analysis using DMRs involved in the BCclassifiers. Figure S7. The predictive performance of transcriptome-based two-step classifier. [file 12864_2023_9471_MOESM1_ESM.pdf]

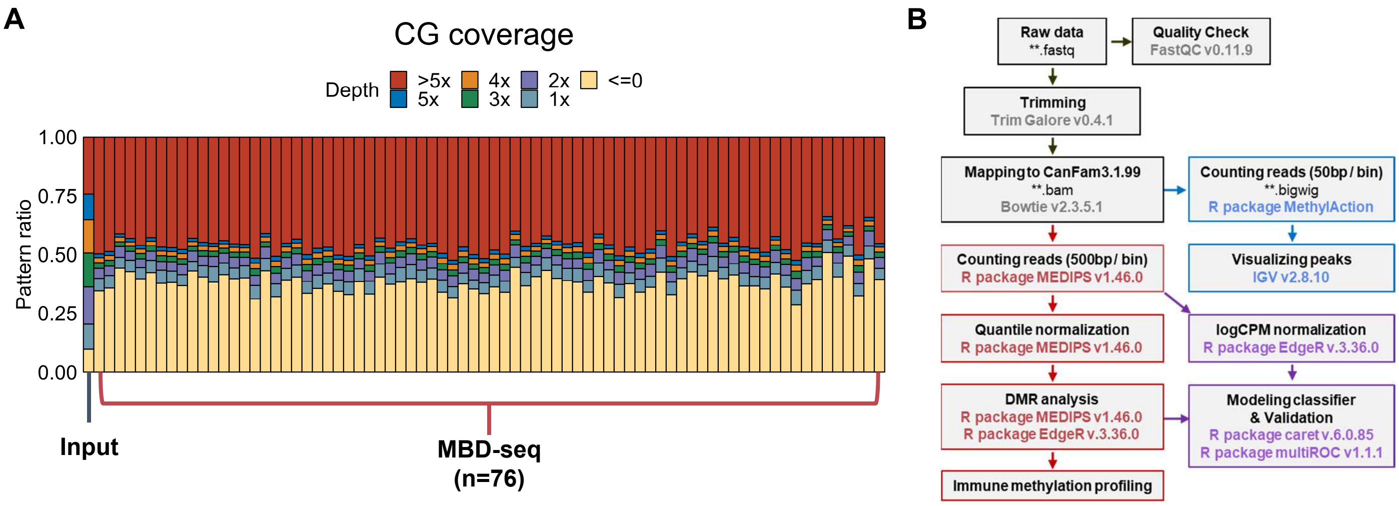

Figure S1. Quality check and processing MBD-seq data. (A) The CpG coverage according to the read depth is shown as a 100% stacked bar plot. Compared to Input (the first bar), about half of genome CpGs have been covered by reads with high depth (>5x) in MBD-seq. It states that MBD-seq data has been successfully enriched in CpG regions across the 76 PBMC samples. **(B)** The workflow of the MBD-seq data processing. After trimming and mapping to CanFam3.1, MBD-seq data were quantified for DMR analysis, peak visualization, and classifier modeling. The black box indicates data pre-processing (from raw data to mapped reads), the blue box shows data processing for peak visualization, the red box exhibits the process of quantitation and normalization for DMR analysis, and the purple box shows the normalizing counts for the classifier modeling.

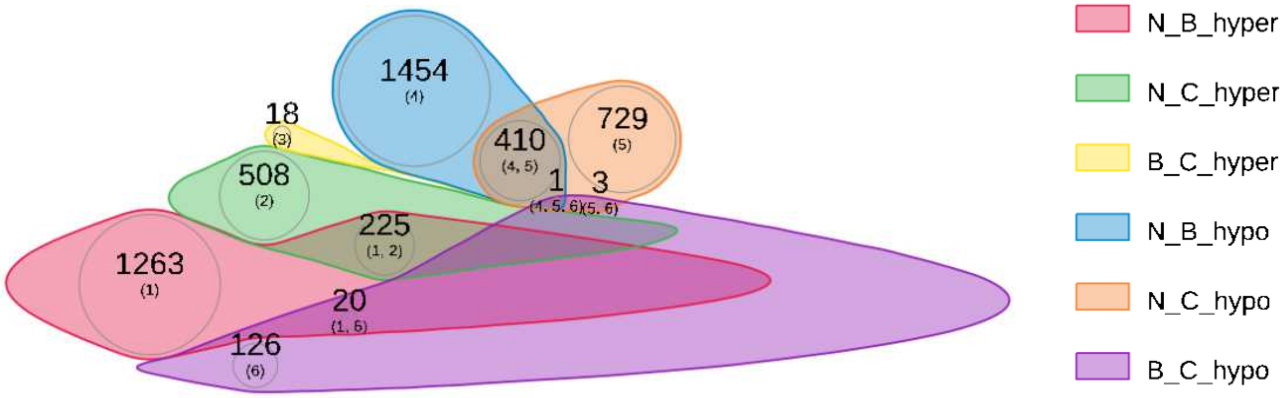

Figure S2. Venn diagram for hyper- and hypo-methylated DMRs. A Venn diagram shows the number of common and unique DMRs identified in each comparison according to the direction of methylation (FDR-adjusted p-value < 0.1 and loFC ≥ ±0.585). There is no common DMR between 'NB_hyper and NC_hypo' OR 'NB_hypo and NC_hyper', which suggests that the methylation pattern in Benign is similar in Carcinoma PBMCs.

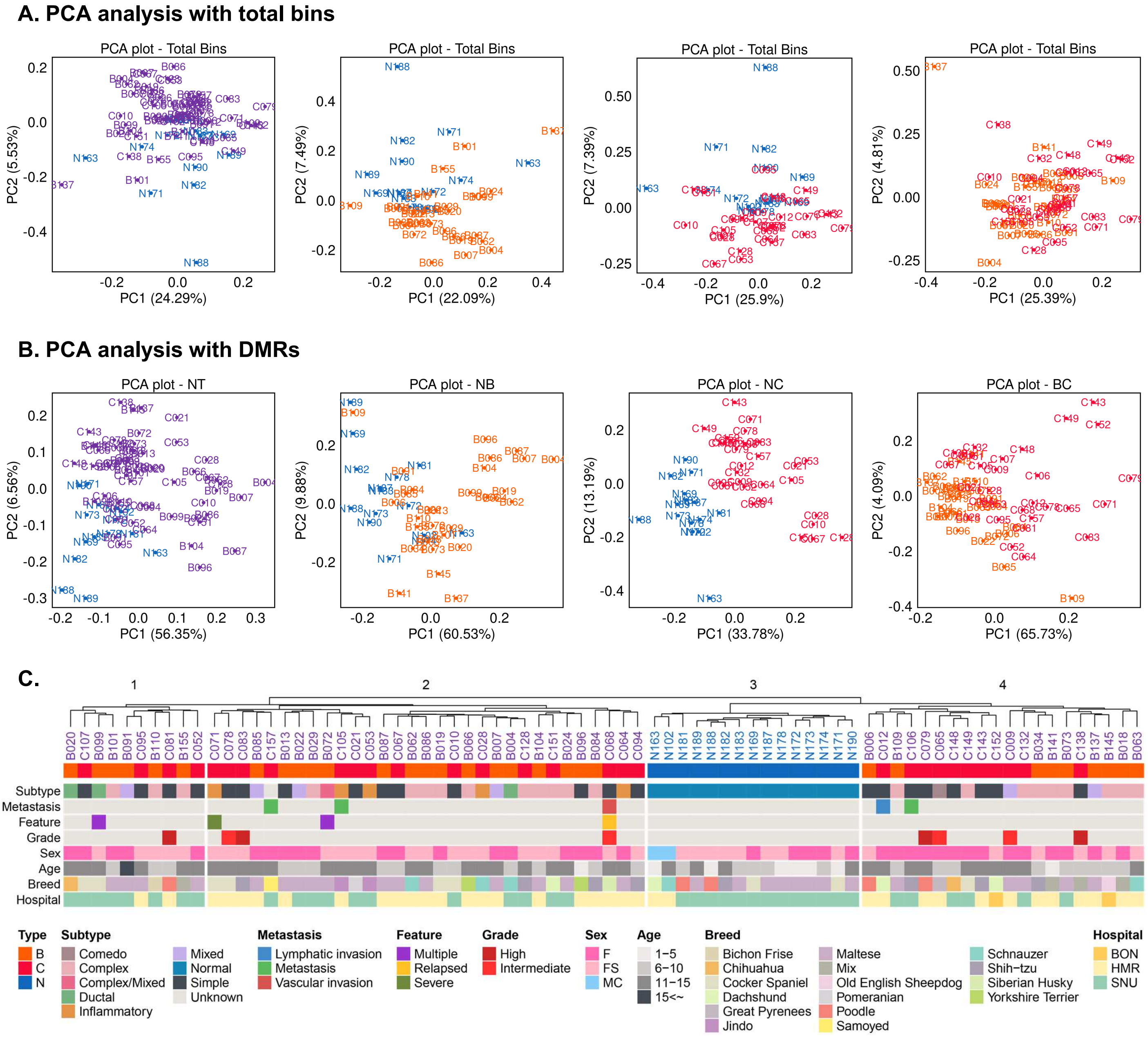

Figure S3. Unsupervised and supervised clustering between comparison groups. (A) PCA clustering comparison groups using total bins. **(B)** PCA clustering comparison groups using corresponding DMRs. Unlike unsupervised clustering using total bins, supervised clustering using DMRs distinguishes two groups. (N: blue, T: purple, B: orange, C: red) **(C)** A total of 2,840 DMRs were identified through a comparison of normal and tumor PBMC samples (|Fold Change| ≥ 1.5 and adjusted p-value (FDR) < 0.1), and subjected to hierarchical clustering to examine the effects of various sample variables. Each column represents a different sample, while each row represents a variable of the samples, including subtype of tumor, metastasis, tumor feature, grade, sex, age, and others.

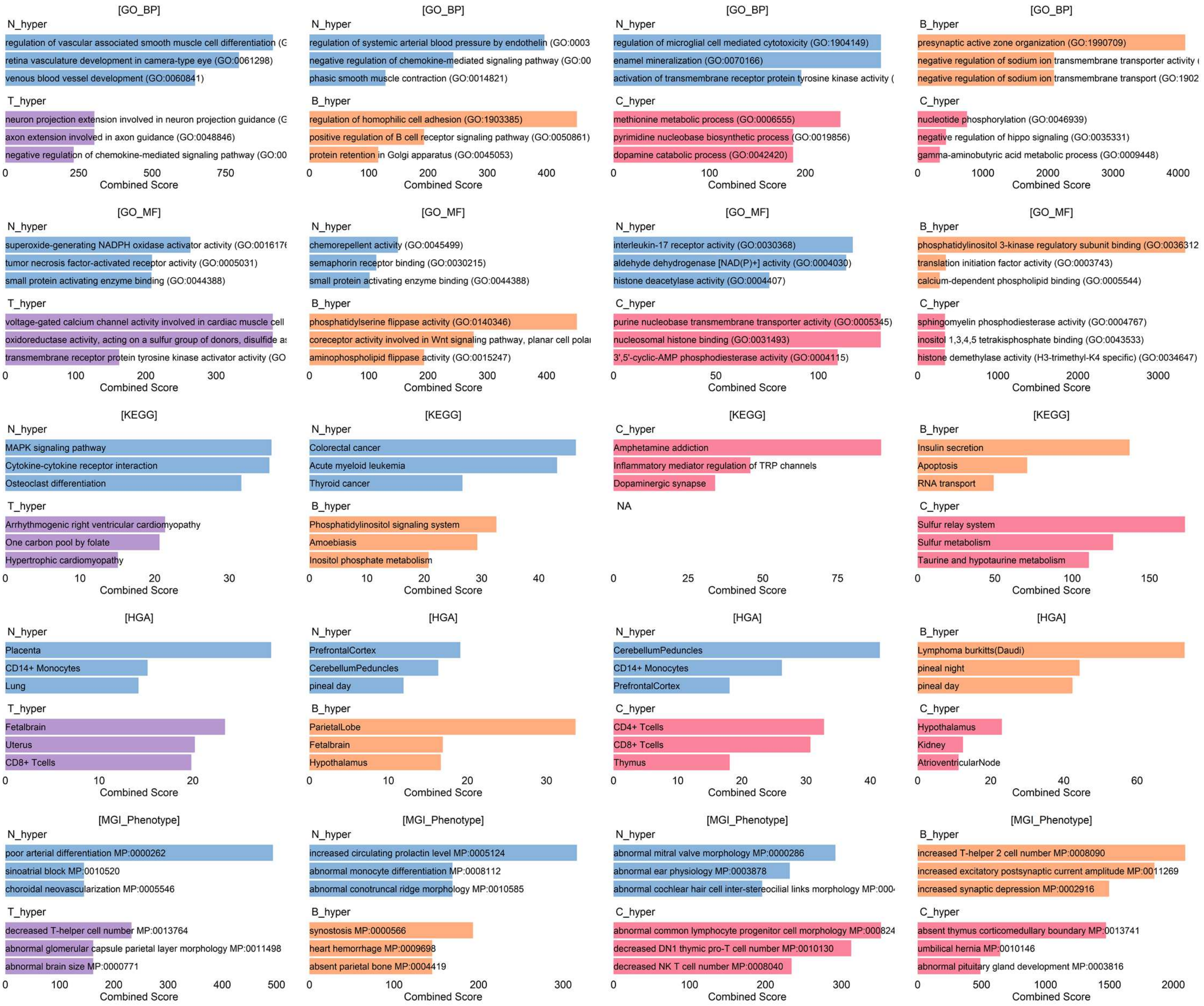

Figure S4. Enriched terms ranked in the Top 3 by combined score according to comparison groups. The top three terms are shown based on the combined score, the unit used in EnrichR [19]. Terms enriched in Gene Ontology (both Biological Process and Molecular Function), KEGG pathway, Human Gene Atlas (HGA), and MGI mammalian phenotypes (MGI_Phenotype) are shown.

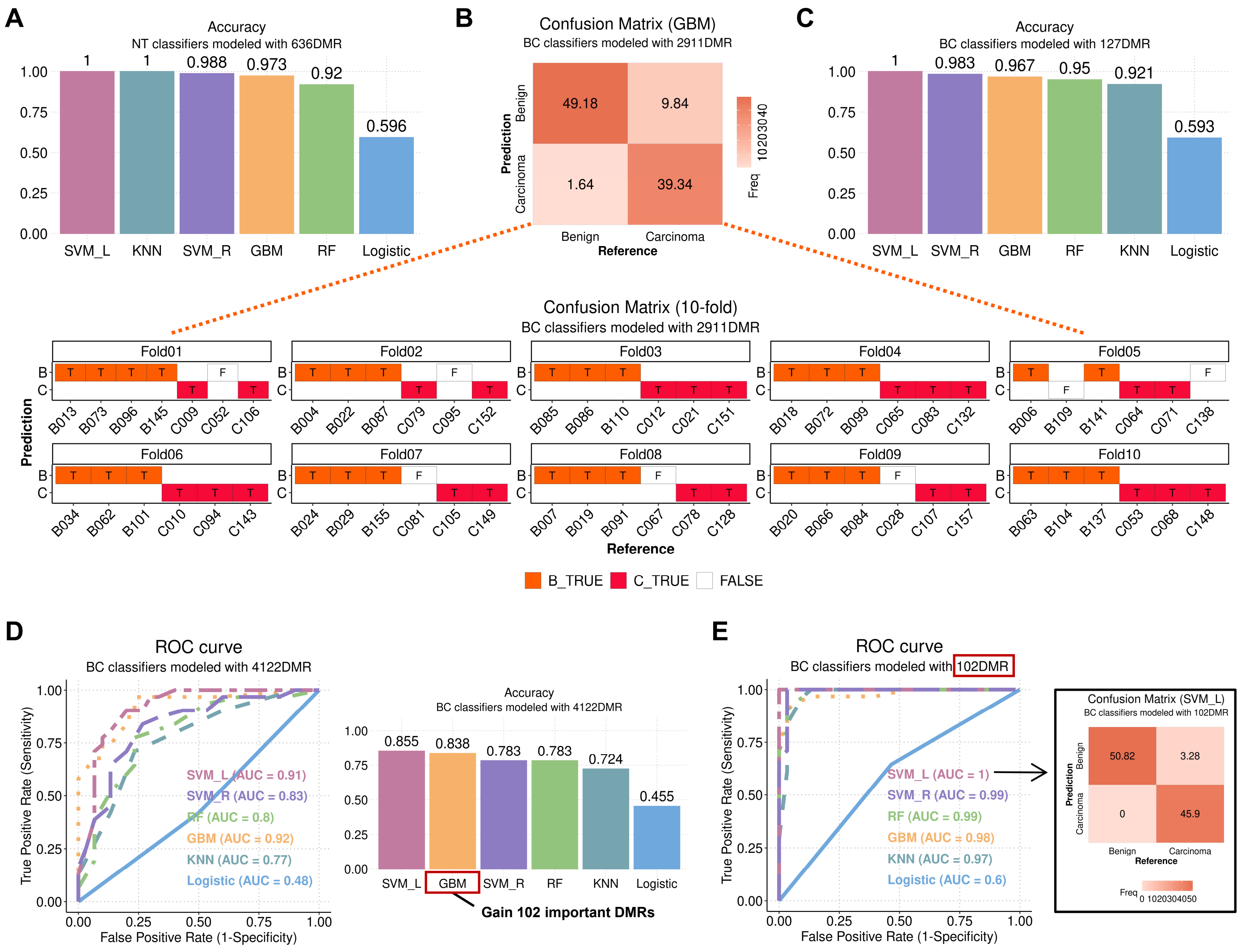

Figure S5. Evaluating the accuracy and predictive performance of the two-step classifier. (A) Classifying accuracy of NT classifiers generated by six ML algorithms. **(B)** Heatmap of the confusion matrix (left) for discriminating Carcinoma from Benign in the GBM-based BC classifier. The confusion matrix for 10-fold cross-validation (right) shows the prediction results for six test samples in each fold. **(C)** Classifying accuracy of BC classifiers generated by six ML algorithms. **(D-E)** BC classifier modeling using 4122 DMRs (FDR < 0.1) is performed in parallel with Fig. 5F-G. The ROC analysis shows the performance of BC classifiers.

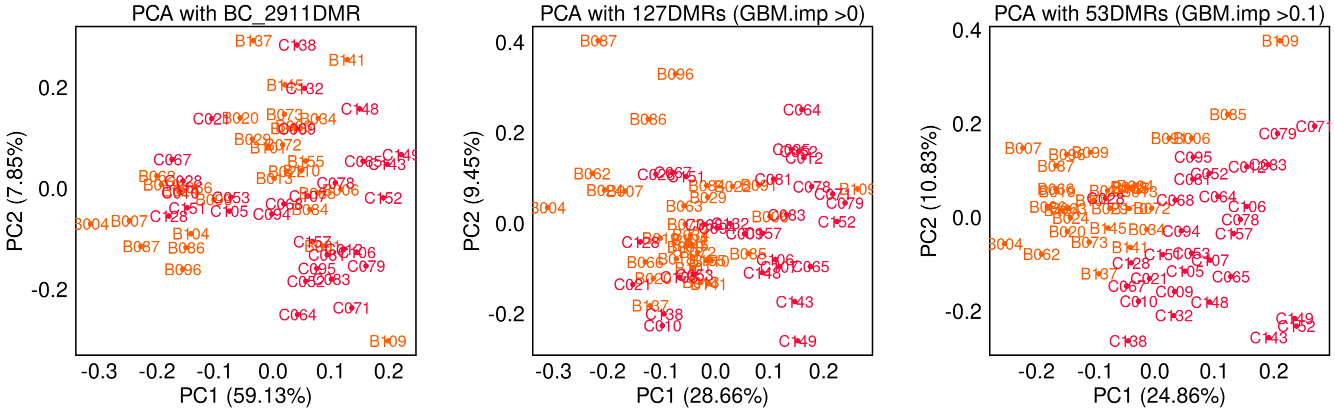

Figure S6. PCA analysis using DMRs involved in the BC classifiers. PCA analysis of 31 benign (orange) samples and 31 carcinoma samples (red) using 2911 DMRs (total DMRs involved in the early BC classifier), 127 DMRs (feature importance scored by GBM upper 0 used for generating the final BC classifier), and 53 DMRs (among 127 DMRs, feature importance upper 0.1). DMRs with high feature importance divide the two groups better, so the feature importance is relevant.

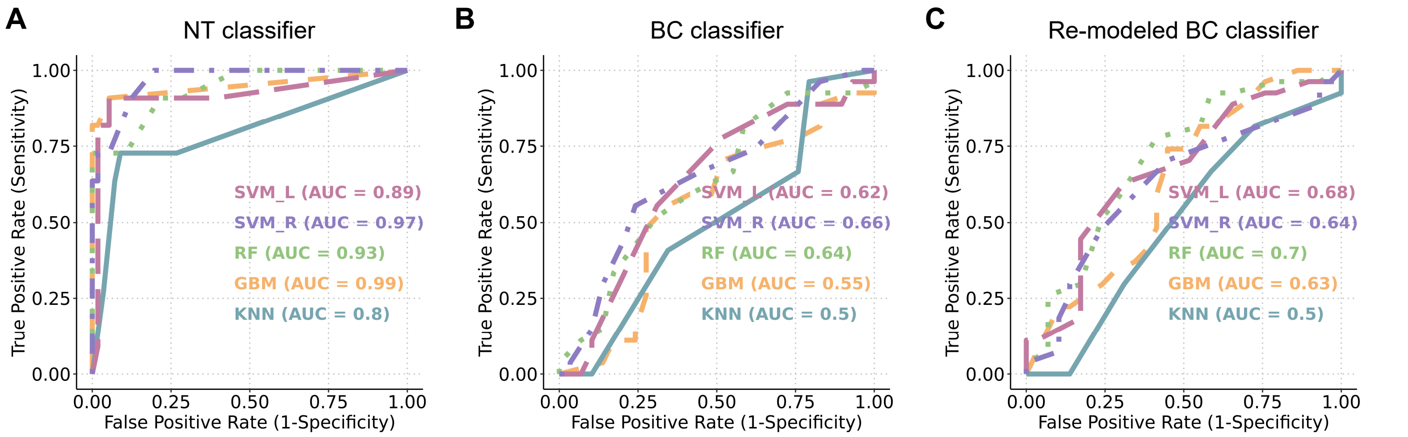

Figure S7. The predictive performance of transcriptome-based two-step classifier. (A-B) The ROC curves of the NT and BC classifiers are shown, established using SVM_L, SVM_R, RF, GBM, and KNN. The right-bottom area under the curves represents the AUC values. The NT classifier was established using 34 genes differentially expressed in benign and carcinoma versus normal PBMCs, while the BC classifier was modeled with 2,181 DEGs differentially expressed in benign versus normal PBMCs as well as carcinoma versus normal PBMCs. **(C)** The ROC curves of the re-modeled BC classifiers using the 1,372 genes did not show improved performance compared to previous BC classifiers, unlike the methylome-based BC classifier.
